# Factors That Influence Climate Change-Related Mortality in the United States: An Integrative Review

**DOI:** 10.3390/ijerph18158220

**Published:** 2021-08-03

**Authors:** Ruth McDermott-Levy, Madeline Scolio, Kabindra M. Shakya, Caroline H. Moore

**Affiliations:** 1 M. Louise Fitzpatrick College of Nursing, Villanova University, Villanova, PA 19085, USA; 2 Department of Geography and the Environment, Villanova University, Villanova, PA 19085, USA; mscolio@villanova.edu (M.S.); kabindra.shakya@villanova.edu (K.M.S.); 3 Georgia Baptist College of Nursing, Mercer University, Atlanta, GA 30341, USA; moore_ch@mercer.edu

**Keywords:** climate change, mortality, integrative review, climate change-related mortality

## Abstract

Global atmospheric warming leads to climate change that results in a cascade of events affecting human mortality directly and indirectly. The factors that influence climate change-related mortality within the peer-reviewed literature were examined using Whittemore and Knafl’s framework for an integrative review. Ninety-eight articles were included in the review from three databases—PubMed, Web of Science, and Scopus—with literature filtered by date, country, and keywords. Articles included in the review address human mortality related to climate change. The review yielded two broad themes in the literature that addressed the factors that influence climate change-related mortality. The broad themes are environmental changes, and social and demographic factors. The meteorological impacts of climate change yield a complex cascade of environmental and weather events that affect ambient temperatures, air quality, drought, wildfires, precipitation, and vector-, food-, and water-borne pathogens. The identified social and demographic factors were related to the social determinants of health. The environmental changes from climate change amplify the existing health determinants that influence mortality within the United States. Mortality data, national weather and natural disaster data, electronic medical records, and health care provider use of International Classification of Disease (ICD) 10 codes must be linked to identify climate change events to capture the full extent of climate change upon population health.

## 1. Introduction

Global atmospheric warming leads to climate change that results in a cascade of events and increases environmental exposures and amplifies health and social vulnerabilities that influence human mortality [1,2]. Over the past 50 years, climate change has caused global temperatures to increase and severe weather events to become more common [3,4]. Each year, extreme heat kills more people in the United States (U.S.) than storms, floods, and lightning combined, making it one of the most lethal natural events [5,6].

United States and international agencies have noted that climate change will have an impact on human health that can influence mortality. The Fourth National Climate Assessment [7] noted that climate change increased health risks of Americans and made already vulnerable and marginalized groups such as children, older adults, people of color, and low-income communities at greater risk of mortality. Those impacts include heat waves, wildfires, poor air quality, infectious diseases (vector-, food- and waterborne), and extreme water events such as flooding. The United Nations Intergovernmental Panel on Climate Change (IPCC) reported that the human toll from climate change globally will increase mortality from heat-related and ozone-related events as well as vector-borne diseases [2]. Extreme weather events such as severe precipitation and flooding have become more common and can amplify the presence of water-borne and vector-borne diseases [8,9].

The human toll of climate change extends beyond the effects of extreme heat. There is a relationship between extreme cold events from ocean warming in the Arctic and weakening of the Arctic jet stream causing cold air to move further south in the Northern Hemisphere [10]. The frequency of wildfires and droughts will increase due to changes in temperature and weather patterns caused by climate change [7]. Wildfire smoke exposure has been associated with an increased risk of respiratory disease, primarily worsening of asthma and chronic obstructive pulmonary disease (COPD), as well as a significant increase in all-cause mortality [11]. Furthermore, droughts decrease the amount and flow of water in an area and this can lead to increased pathogens as warm and stagnant waters are favorable conditions for their growth [3]. The greater prevalence of droughts and wildfires coupled with an increase in greenhouse gas emissions has had serious negative effects on air quality in the United States which has been linked to an increase in mortality related to pulmonary and cardiovascular diseases [7,12].

While most research has focused on how specific aspects of climate change (heat waves, extreme weather events, flooding, etc.) have affected mortality, few studies give an in-depth analysis of the scope of climate’s effect on factors that influence climate change-related mortality [3,13,14]. It is important to have a comprehensive understanding of the collective factors that influence climate change mortality to understand the impacts on human health, thus we are examining the climate change-related factors that lead to mortality. Mortality is used as the measure of climate change impacts because mortality is an indication of risk and severity of an exposure [15], such as climate change. Therefore, we conducted an integrative review of the peer-reviewed literature to identify the factors that influence climate change-related human mortality in the United States in the past decade (2010–2020). The review was limited to the U.S. to reduce confounding of other factors that influence mortality such as country specific climate change adaptation and resilience, health systems, and access to health care. The period of 2010–2020 was selected to provide the most recent decade experiencing the impacts of climate change.

## 2. Methods

**Figure 1:**
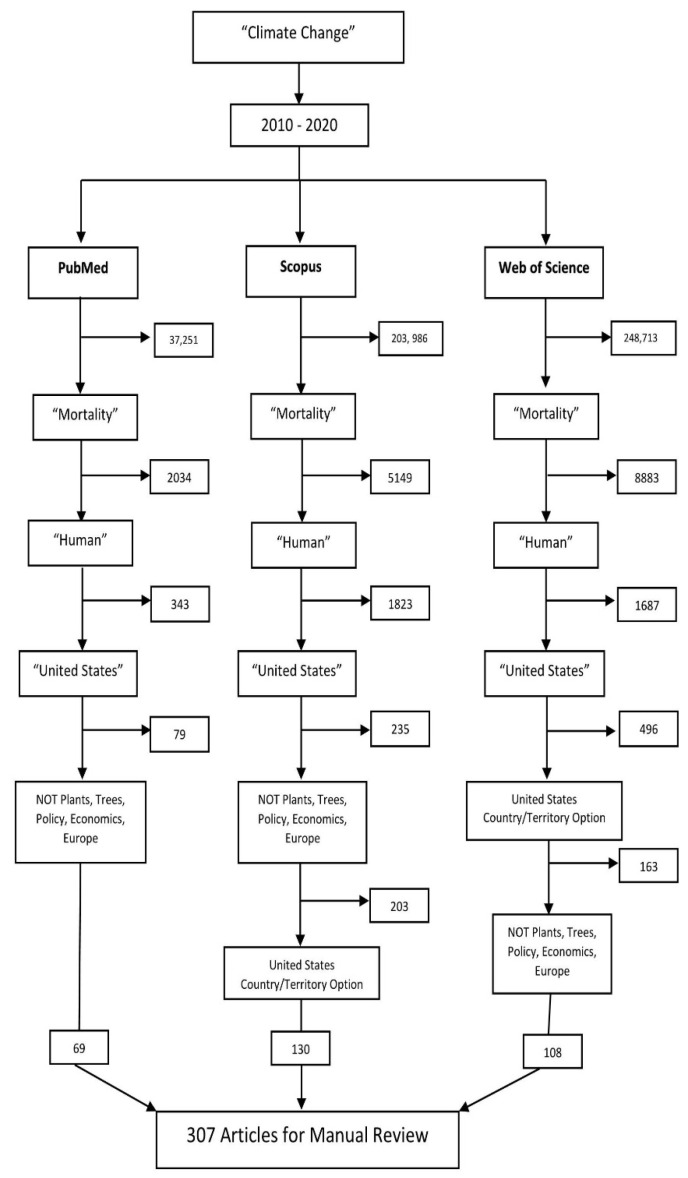
The number of articles retrieved from three databases: PubMed, Scopus, and Web of Science using the term “Climate Change”. The keywords used for filtering are shown inside the quotes.

**Figure 2:**
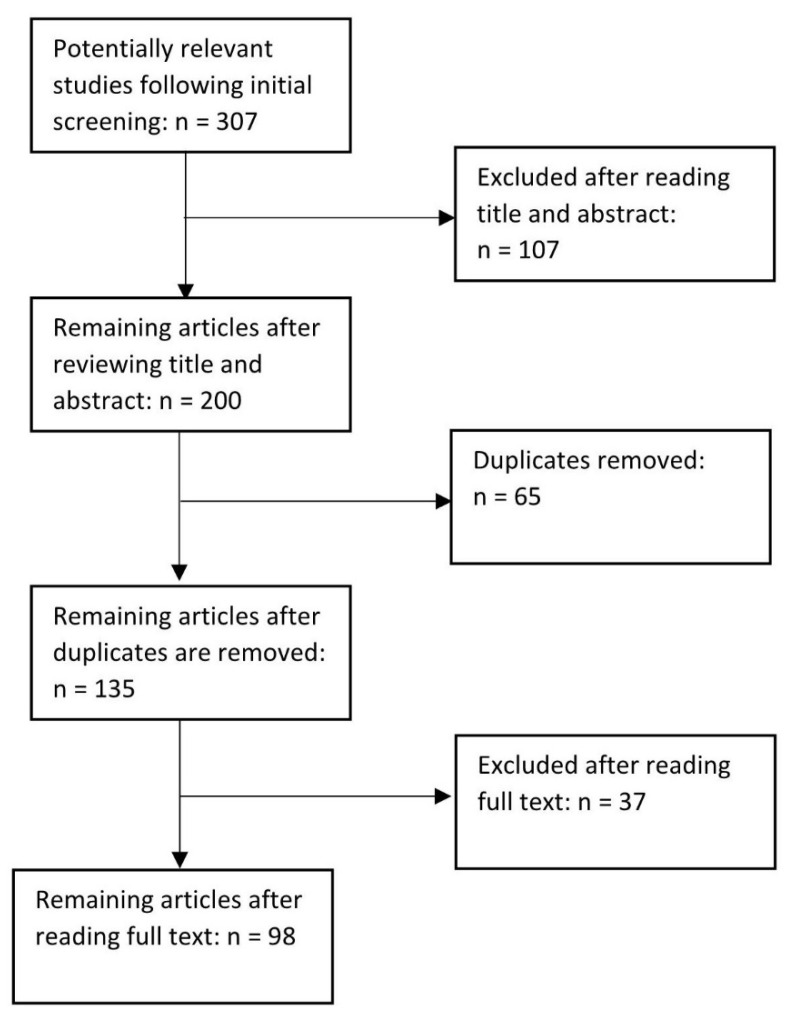
Final number of articles included after the review for relevance and duplicates.

This integrative review was guided by Whittemore and Knafl’s [16] framework which includes (1) problem identification; (2) literature search; (3) data evaluation; (4) data analysis; and (5) presentation. Problem identification was to address the question: What are the factors that contribute to climate change-related mortality? The literature search strategy included three databases—PubMed, Web of Science, and Scopus—with a filter by date, country, and keywords. The search terms were “Climate change”, “Mortality”, “United States”, and “Human” (Figure 1). We also excluded any results containing the keywords “Plants”, “Trees”, “Europe”, “Economics”, and “Policy”. We further limited our search to literature from the United States, published between 2010 and 2020 in English. The original search yielded 69 articles from PubMed, 130 articles from Scopus, and 108 articles from Web of Science. Next, the titles and abstracts of the resulting articles were read and any articles that did not match purposes of identifying the factors that influence climate change-related mortality in the United States in the past decade were removed. Most articles excluded at this stage were related to animals, air pollution, economics, and other countries. Articles that linked climate change and mortality with air quality or air pollution were included; those that only addressed only air quality or air pollution were removed. After this screening, 66 articles from PubMed, 74 articles from Scopus and, 60 articles from Web of Science remained. These articles were then uploaded into a Zotero (a free and open-access reference management software program, version 1, Corporation for Digital Scholarship, Vienna, VA, USA), library to determine duplicate articles from the search. After all duplicates were removed, 135 articles remained. Data analysis included four researchers reading all the 135 abstracts and deciding to include or exclude each paper. Articles were removed if they did not meet any of the original search criteria or if they did not address climate change-related mortality. Any questions about the literature were discussed among the researchers. As with integrative reviews, theoretical or review articles were included since they contained important background information about climate mortality and often made connections between quantitative research and climate change mortality. Thirty-seven articles were excluded after this phase, leaving 98 articles that were included in the review to identify factors that influence climate change mortality in the United States. The final step of this review, the presentation phase is presented in the results section, Figure 1 and Figure 2, and Appendix A at the end of this article.

## 3. Results

Our review yielded two broad themes in the literature that addressed the factors that influence climate change-related mortality. The broad themes are environmental changes and social and demographic factors. Within each theme, there are subthemes that further describe the analysis of this review. 

### 3.1. Environmental Changes from Climate Change

#### 3.1.1. Ambient Temperatures

##### Heat

Climate change-related heat events pose the greatest risk to human mortality [17]. Geographic location, timing of the heat event [18,19], socioeconomic status, occupation, and age all influence a population’s ability to adapt to extreme heat [20,21,22,23,24,25,26]. It is well known that extreme heat and other impacts of climate change can exacerbate already existing conditions and lead to premature death [27,28,29,30,31,32]. In fact, Guo and colleagues [33] used modeling to predict overall excess heat-related mortality from climate mitigation and adaptation and projected that the U.S. 2031–2080 population growth would be 34.5% with a low climate response, 54.2% with a moderate response, and 76.2% with strong climate action. In other words, without heat-related climate strategies, U.S. communities will experience a slower growth rate because of excess heat-related mortality. Yet, quantifying this is challenging given the underlying comorbidities [34]. While examining those who died during heat waves, Wilson et al. [32] noted that 47% of the deceased had cardiovascular disease, 27% psychiatric illness, 16% neurologic disorders, 10% pulmonary disease, 9% endocrine disorder, and substance abuse. There are increased instances of death due to congestive heart failure, heart attack, stroke, diabetes, COPD, and pneumonia on extremely hot days [32,35]. Additionally, higher temperatures were associated with higher rates of all-cause mortality between 1999 and 2011 in Rhode Island [36].

Older adults and those with chronic illnesses are especially vulnerable to heat-related mortality [37]. Heat susceptibility can be influenced by medication regimen, as some medications have been shown to affect the body’s ability to thermoregulate and physiologically adapt to heat [38]. An increased ambient temperature of 1 °C has been associated with a 1.0% higher death rate in the summer [25]. A 1.5 °C increase in yearly temperature is projected to lead to 1605 (95% CI: 1430, 1776) excess deaths annually [39]. An examination of heat-related deaths in 40 U.S. cities found a decrease in extreme heat-related excess deaths from the 1975–1995 to the 1996–2004 period. The reduction was attributed to better heat event forecasting, public health education, and warning systems [40]. Seasonality and region also play a role in heat-related mortality. For example, during the summer months in Florida (May–October), there was an increase in work-related (25/100,000) and non-work-related deaths (158/100,000) during the 2005–2012 study period [21]. Further, exposure to large temperature variability was found to lead to an increased risk of mortality [41]. Climate change-related heat mortality is further described in the social and demographic factors that influence mortality.

##### Cold

Although there are few studies examining the impacts of cold weather on mortality, climate change has increased the intensity of winter storms and extreme cold weather effects on human health [42]. Colder winters are expected in the U.S. Midwest and Northeast [43]. Increasing winter temperatures of 1 °C can lead to a 0.59% (95% CI: 0.37%, 0.81%) increase in annual mortality [25]. The relationship between age and cold-related mortality is inconsistent. Older adults, and those with cardiovascular and cerebrovascular disease have an increased risk; however, there is also evidence of cold-related mortality in younger groups [43]. Power outages during cold events were found to have a positive association with all-cause mortality and non-external cause mortality [44]. Increases in outdoor physical activity (snow shoveling) and respiratory infections also contribute to mortality. Cold air suppresses the immune system, can cause bronchoconstriction, and the air is less humid [43], leading to pathogens floating more easily in close indoor spaces. Cold-related mortality has been observed on the same day of the cold event and lasting as long as 24 days after cold weather [43]. In addition, Shi et al. [25] observed that with a temperature increase of 1 °C, there was a 3.49% (95% CI: 3.08%, 3.90%) decrease in mortality in the winter in the Southeastern U.S. This was attributed to the region having more variable winter and summer temperatures, which made the 1 °C increase health protective. With warming winters, 10 U.S. metropolitan areas found that future temperature estimates correlated with decreased rates of cold-related deaths. However, in eight of the ten metropolitan areas, the greater mortality risk with heat negated the health benefit of warmer winters [45]. Conlon and colleagues [43] noted that smaller, rural communities had a greater risk of cold-related mortality. Without consideration of climate adaptation, any benefit of reduced mortality during warmer winters was outweighed by the premature temperature-attributable deaths during summer heat events [46,47,48,49]. Annual excess co-morbidity-related mortality (from non-communicable diseases) in New York City during 1997–2013 was found to be eleven times greater from extreme heat than average annual hypothermia-related mortality [37]. Additionally, Barnett et al. [42] found that a reduction in extreme cold events and cold waves later in the winter were associated with lower mortality.

Despite the gains in mortality from warmer winters, Hansel et al. [20] reported that individuals over 65 years old with COPD had a 19% increased risk of dying on cold days. Cold temperatures are associated with mucous hypersecretion, bronchoconstriction, inflammation, and increased risk of exacerbation of COPD [20]. Extreme cold was also associated with an increased risk of acute myocardial infarction (MI) [50].

##### Accidents and Injury Related to Ambient Temperatures

Accidents related to warmer winters have been observed in Alaska, an Arctic state. Alaska has warmed at more than twice the rate of the rest of the U.S. This warming has led to thinning of ice and increased risk in falling-through-the-ice (FTI) events. More than 35% of 307 Alaskan FTI events involved a fatality, most commonly from drowning. Nearly 5% of people were not found and were presumed dead, but additional reports confirming drowning mortality were lacking [51]. Power outages, which are indirectly associated with extreme weather events from climate change, were associated with a 122% (CI: 28%, 287%) increase in accidental deaths during the 2003 New York City blackout [52] and increases in all-cause and non-external cause mortality [44].

There is evidence of a relationship between higher temperatures and fatal motor vehicle accidents. Hot days were associated with driver irritability, drowsiness, and missing signals that affected driver performance [53]. More fatal crashes of adults on rural roads occurred on days of higher heat with medium to high solar radiation and without precipitation than on non-heat days [53]. Additionally, obese drivers (body mass index, BMI ≥ 30 kg) had 3.0% (95% CI: 0.0%, 6.0%) higher odds of fatal traffic crashes on heat wave days than non-heat wave days [53]. A review of injury deaths from 1980 to 2017 found that fatal transport accidents and drownings were highest during the summer. However, seasonal patterns were not as clear for other forms of injury deaths such as suicides, falls, and assault [39].

#### 3.1.2. Air Quality

Atmospheric photochemical reactions are likely to be enhanced with increasing temperatures. Most importantly, ground-level ozone and particulate matter (PM) concentrations will be impacted. Increased ozone or particulate matter concentrations have been linked to increased mortality related to pulmonary and cardiovascular disease [4,9,54,55,56]. Mortality related to elevated PM_2.5_ is amplified in populations that are overweight/obese and have metabolic syndrome, and/or subclinical inflammation [57]. It is estimated with a greenhouse gas (GHG) concentration under representative concentration pathway (RCP) 8.5 and increased arid conditions in the U.S. Southwest, increases in PM_2.5_ will be responsible for an increase in mortality of 120% by 2050 and 230% by 2090 from cardiopulmonary-related deaths in adults 75 years old and older [58]. However, under RCP 4.5 GHG and emissions reductions, an estimated 16,000 (CI: 11,700, 20,300) deaths from PM_2.5_ per year could be prevented by 2050 [59].

Ground-level ozone formation is favored with hotter temperatures and stagnant conditions while precipitation will impact PM concentrations [12]. Regions that experience more sunshine and poorer air quality will be at greater risk for ozone-related mortality [60,61]. There is a wide range of data related to mortality and morbidity due to changes in ozone concentrations from climate change. The climate change air quality models have predicted 600 to 2500 cases of nonaccidental mortality related to climate change that could be avoided from the changes in ozone concentrations [61]. Chang et al. [62] estimated a 0.01% increase in mortality due to elevated ground-level ozone concentrations related to future climate change. Future ozone concentrations are expected to increase by 0.43 ppb as reported in the prediction interval (PI) (95% PI: 014–0.75) in the period 2041–2050 compared to in 2000 [62]. There are also predictions of a 4 ppb increase in summertime daily maximum (8 h average) ozone concentration in the eastern United States in the 2050s (referenced in [12]). Climate change may result in summer daily maximum (8 h average) ozone concentrations changing by 3% and annual PM_2.5_ concentrations increasing by 3 to 6% of mean annual PM_2.5_ concentrations [12].

#### 3.1.3. Drought and Wildfires

Mortality from drought is typically an indirect factor resulting in long-term secondary exposures such as increased airborne dust, wildfire smoke, food insecurity, and malnutrition leading to premature death. Chronic psychological stress from drought is associated with behavioral and physiological responses, including hemodynamic, endocrine, and immunological dysfunction that increase risk of cardiovascular and upper respiratory disease, and suicide [63].

There is growing evidence of associations between wildfire smoke exposure and all-cause mortality [11]. Burn injuries, posttraumatic stress disorder (PTSD), and acute exacerbation of respiratory conditions such as asthma, decreased lung function, chronic obstructive pulmonary disease [3], and cardiovascular outcomes [11] have been associated with wildfire mortality. A limitation in determining the impact on health and mortality from wildfire smoke is that wildfires tend to occur in rural areas where there are fewer or no air pollution monitoring networks to make an association between air quality and mortality. Reid et al. [11] noted that there are too few studies and inconsistent findings to determine an association between wildfire smoke and cardiovascular mortality, despite the evidence that PM is associated with increased cardiovascular deaths.

#### 3.1.4. Precipitation and Flooding

Flooding is associated with surface and drinking water contamination from overflowing sewers, agricultural run-off, and degradation of infrastructure. Infrastructure degradation can disrupt public health and health care access [3,4,64]. Carnes et al. [4] cited a Centers for Disease Control and Prevention study that noted that gastroenteritis-associated deaths rose from approximately 7000 to more than 17,000 yearly over the past 8 years, with 83% of those deaths occurring in older adults (> 65 years old). Indirect impacts of flooding that may have associated mortality but may not be attributed to climate change. These include exposures to mold [3] or other pathogens (such as Legionella) [64], injury from the flooding event, or failure to rescue from an overwhelmed health system during a flooding emergency [4].

#### 3.1.5. Infectious and Vector Borne Disease

More precipitation from climate change may block drainage systems and produce stagnant water; this plus warmer air temperatures can create a breeding ground for diseases carrying insects. In fact, the overall number of insects is expected to rise globally [9,65]. Raffa et al. [65] found that dengue along the Texas River increased 2.6% a week after a 1 °C increase and increased 19.4% at 18 weeks after a 1 °C increase in sea surface temperature. Climate change could affect the frequency and expand the geographical range of diseases [66,67]. For example, the free-living ameba *Naegleria fowleri* that can cause primary amebic meningoencephalitis was detected in water and sediment samples during above normal warm waters in Minneapolis, Minnesota, approximately 500 miles north of the previously reported location in Missouri [66]. Furthermore, pathogens may develop resistance to antibiotics with increasing temperatures [68].

#### 3.1.6. Mental Health Related to Environmental Changes

Individuals who personally experience extreme weather events such as a heat wave, hurricane, wildfire, prolonged drought, or falling through ice may experience distress, anxiety, and other emotional consequences [13,51]. Berman et al. [63] found a 15% increase in suicide among rural male farmers when severe drought was present. The uncertainties of climate change in the future can cause individuals chronic stress and anxiety [13,63]. Chronic stress can affect one’s behavior and psychological responses, depression, anxiety, and hemodynamic stability [63]. A study reported that increase in temperature in U.S. cities might affect human wellness [69].

### 3.2. Social and Demographic Factors

#### 3.2.1. Geographic Factors

Climate change, with the accompanying increase in temperatures, does not affect different geographic regions equally [70]. Locations with lower air conditioning usage and milder weather are more susceptible to heat-related mortality as there typically is not residential air conditioning and no way to cool the indoor environment [27]. For example, people who live in locations with cooler summer temperatures such as the Pacific Coast have a greater relative risk for heat-related mortality than people who live in places like the Gulf of Mexico with warmer summers and moist subtropical climates where residential air conditioning usage is more common [71]. Heat-related mortality in the United States has been found to be higher in northern latitudes as compared to southern, suggesting that areas regularly exposed to higher temperatures have already begun to adapt effectively [18,72,73,74]. This could be because people residing in lower latitude regions physiologically and behaviorally were more prepared to respond to warmer temperatures by drinking more water or using air conditioning [21].

People living in northeastern U.S., especially Massachusetts, New Jersey, and New York, are also more vulnerable to heat-related mortality as this area is more densely populated and contains more metropolitan areas than the southern regions [14,75]. During a heatwave, an increase in average temperature of 1 °F was associated with a 4.39% increase in the relative risk of mortality in the Northeast and a 3.22% increase in the Midwest, making the Northeast the region with an average of 2.50% greater risk of daily mortality during a heatwave [18].

Warmer temperatures were found to have a greater effect on mortality in higher latitudes in the U.S. where the mean temperatures were lower than in lower latitudes which had higher mean temperatures [26]. The southeast region of the U.S. along the Atlantic Ocean and Gulf of Mexico will be affected more from climate change-related events, such as sea level rise and subsequent land loss, frequent heat events, hurricanes, and drought [76].

#### 3.2.2. Rural

Rural areas are also at risk of heat-related mortality [77] and wildfires [11]. The ability to quantify the adverse health effects caused by climate change is limited in many rural areas due to lack of available data and interest, potentially leaving many vulnerable groups unaccounted for [24]. Furthermore, rural communities frequently do not have heat warnings or heat-protective community programs, and outdoor agricultural work is a leading industry; thus, placing rural dwellers at greater risk to succumb to heat. Like rural areas, even smaller more spread-out cities lack heat adaptations and warning systems as the risk of heat exposure is underestimated when compared with more densely populated urban centers [78]. The least populated areas of Illinois had average annual hospitalization rate due to heat stress of 1.16 hospitalizations per 100,000 people, while metropolitan areas had 0.45 hospitalizations per 100,000 people [79].

Drought is a major contributor of mortality in rural areas. Sheridan and Kalkstein [80] reported that there was four-times greater risk of mortality for rural than for urban counties during severe periods of drought. Drought-related suicides and mental health problems are most severe among rural populations [63,80].

#### 3.2.3. Urban

Urban areas have unique risk related to population density and the built environment. Urban heat islands (UHI), which occur when urban areas have warmer temperatures than their rural surroundings due to the heat trapping abilities of urban development, [81,82] intensify heat by 1.9 °C ± 0.7 °C [37,45]. Climate change exacerbates UHIs and is responsible for increasing morbidity and early mortality [72,78]. The impact of UHIs is associated with a 2.2% increase in mortality; however, with an RCP 8.5 scenario, mortality is projected to increase to 4.3% by the end of the century [83]. Urban areas are also projected to experience greater respiratory-related hospitalizations with increased mortality by the end of this century [84]. Low-income urban residents [85] in cities with mild summers and fewer residents with air conditioning units experience greater mortality during heat waves [27]. Additionally, those living in more urban areas were more likely to suffer from an acute myocardial infarction on extremely hot days, measured by a hazard ratio (HR) of 1.48 (95% CI: 0.88, 2.49), than those living in less densely populated areas (HR = 0.81 (95% CI: 0.61, 1.08)) [50]. Heat stress, measured by heat stress index (temperature and humidity index), affects all populations regardless of urban or rural settings [77] and mortality related to heat are not limited UHIs [85].

#### 3.2.4. Influence of Gender

Gender plays a role in climate change mortality. Men are exposed to more outdoor activities and work in conditions above 27 °C than women (56% of male workers men compared to 46% of female workers) [86] and men more frequently (age 15–64 years old) participate in more strenuous outdoor activities [87]. Despite this, the relationship between gender and heat-related mortality varies by cause of death and region [72]. Some studies have not found any gender differences, while others have found that the effect of heat mortality was greater for females. 

Pre-existing conditions and race play a role for women’s risk of heat-related mortality [28,35]. Across all age groups, women with a history of cardiac disease and stroke had a greater relative risk for deaths with higher ambient temperatures [28]. Older (≥65 years old) women with COPD or asthma and older men with respiratory disease (non COPD/asthma) were at greater risk from heat-related death [28]. Non-White women had a higher risk of mortality from heat and cold [35]. Despite the documented risks for women, on extremely hot days (>35 °C) in Michigan, men had 1.12 times (1.01, 1.25) greater risk of dying than women [64]. However, when compared to males, female workers were 32 times less likely to die from a heat-related illness [88].

Men are more likely to succumb to climate change-related accidents and injury. It is estimated that 1605 (95% CI: 1430, 1776) excess annual deaths from injury could be caused by 1.5 °C increase in annual temperatures [39]. 84% of these deaths are projected to be males [39] with the excess death a result of transport injuries (resultant risk 739; 95% CI: 650, 814) and suicides (resultant risk 540; 95% CI: 445, 631) [39]. The risk of death of heat or dehydration is greater in young males (maximum ambient temperature, ATmax = 41.1 °C, RR = 1.18) and older females (ATmax = 40.6 °C, RR = 1.05) [28].

#### 3.2.5. Age

The very young (<5) and older adults (>65) are the most vulnerable to the effects of climate change. However, older adults suffer the greatest consequences from heat [4,6,9,11,21,27,73,89,90,91,92,93]. Both young children and older adults are unable to adequately thermoregulate, putting them at increased risk for heat-induced illness [21,94]. Infants, children, and older adults are sensitive to the effects of air pollution [4]. Teens and young adults suffer from heat-related illness from participation in athletics or work-related risks [21]. Additionally, older adults frequently have underlying chronic health conditions [3,17,95]. Schmeltz et al. [96] found that older adults do not consider themselves at risk and may not yield to heat warnings on extremely hot days. Despite the risk of heat-related deaths for sensitive groups, a 10% increase in mortality was observed in all age groups from extreme heat in Washington state [97].

There are increased hospitalizations of older adults on extreme heat days relating to respiratory conditions, circulatory, cardiovascular, cerebrovascular, and all-cause admissions [3,85,98]. Limaye et al. [73] calculated an excess 11,562 deaths in persons over 65 years old following extreme heat episodes during the summer in the Eastern United States. This equates to 20.20 excess deaths per 100,000 persons for older adults compared to 2.40–3.23 per 100,000 for the general population [73]. Emergency department visits due to heat were most frequently work related for young adults (ages 25–29) and non-work related for 16–34-year-old Florida residents during warm months (May–October) [21].

#### 3.2.6. Race and Ethnicity

In the United States, heat-related mortality has a greater impact on racial and ethnic minorities [98,99]. For example, Hispanic males have a higher risk for heat-related mortality among industrial workers in the U.S. than non-Hispanic males [88,98]. A case study in Oklahoma suggested that Black people have the highest risk for heat-related morbidity (0.76 deaths per 100,000) compared to Hispanic (0.35) and White (0.46) populations [87]. As racial and ethnic minorities are already likely to have multiple stressors, the added threat of climate change might make these groups suffer first and worst [13]. A study conducted in Maricopa County in Arizona showed that there was higher climate change vulnerability and more deaths in inner-city neighborhoods than in suburban neighborhoods with higher income and education levels, as well as younger White populations [100].

#### 3.2.7. Occupation

Outdoor occupations that require physical exertion, such as construction and agriculture, make workers more vulnerable to the impacts of heat [98,101]. Agricultural workers had the highest annual heat-related mortality rate at 3.06 per 1 million workers, followed by construction (1.13 per 1 million workers), and waste and remediation services (0.56 per 1 million workers) [88]. Other studies have found that construction workers had greater rates of heat stress-related mortality than other occupations [98]. A review of Occupational Safety and Health Administration (OSHA) heat-related deaths (age range 18–72) found that of 79 deceased workers, 76 were male outdoor workers (agriculture, construction, landscaper, oil and gas, and warehouse) [102]. Additionally, there are ethnic, racial, and gender variabilities with mortality among workers from all industries. Hispanic workers had greater risk of mortality than Black workers (RR = 3.22; 95% CI: 2.5, 4.0, and RR = 1.5; 95% CI: 1.1, 4.4), respectively [88]. The risk of death increased when working alone and heavy work. Roelofs [102] reported that most deaths occurred after three or more consecutive days of temperatures at or above 30 °C. Additionally, although several workers succumbed to the heat during the heat waves, others died on cooler days following the heat event [102].

#### 3.2.8. Socioeconomic Status

Socioeconomically disadvantaged communities have been disproportionality affected by climate change [94]. Low-income communities are often less resilient to the adverse effects of climate change as residents may have difficulty affording or accessing health care and adaptive measures such as air conditioning [72,94,103]. They are also more likely to live in urban areas [4]. For example, daytime surface temperatures were significantly higher in New York City neighborhoods with high rates of poverty [104]. Housing quality also contributes to heat-related mortality risk as neighborhoods with poorer quality housing have been found to have higher mortality rates [104]. Excess heat-related mortality in New York City neighborhoods is related to poverty, air conditioning access, educational attainment, housing, quality, homeownership, land cover, and land surface temperatures [105]. Madrigano et al. [50] found that people in census tracts with less than 14% of families below the poverty line were less likely to suffer from acute myocardial infarction on extremely hot days than people who lived in census tracts with higher rates of poverty.

While most heat-related mortality occurs during or directly after extreme heat events, a large portion of heat mortality in many major cities still occurs on more temperate days, which suggests that measures like greater availability and access to air conditioning in addition to current heat emergency responses for extreme heat events are necessary to decrease vulnerability to heat [42]. Relative risk for heat-related mortality has been reduced with greater use and access to indoor air conditioning systems [21]. A study of heat-related morbidity in Oklahoma found that out of the 95 deaths for which they had data of access to air conditioning, 91 (96%) of the deaths did not use air conditioning [3]. Older adults and people from lower socioeconomic status have lower rates of air conditioning use which puts them at greater risk for heat-related death [50]. Access to air conditioning units as well as electricity prices limit air conditioning use as communities may view it as a luxury instead of a necessary adaptive measure to cope with extreme heat [106].

## 4. Discussion

Of the 98 scientific articles reviewed, the meteorological impacts of climate change yield a complex cascade of environmental and weather events that affect ambient temperatures, air quality, drought, wildfires, precipitation, and vector-, food-, and water-borne pathogens. The environmental changes from climate change amplify the existing health determinants that influence mortality within the United States. We found these factors to be in line with the social and demographic disparities that comprise the social determinants of health. Furthermore, heat-related deaths associated with climate change were most frequently studied. Heat compounds existing morbidity, including mental health risks, and geographic, gender, age, racial and ethnic, occupational, and socioeconomic factors that were found to be associated with higher rates of mortality. As expected, older adults are at greater risk of climate change mortality related to existing morbidities [3] and physiological changes of aging [21,96].

Although a warmer winter may be “mortality protective”, extreme cold presents a risk of acute myocardial infarctions [50] and increased mortality for people with COPD [20]. Additionally, warming winters have led to falling-through-the-ice events with drownings [51]. Despite limited research on mortality related to prolong periods of extreme cold and hypothermia [37,45,46], given the extreme cold events in the southern U.S. during the winter of 2021 with subsequent mortality, this is an area that needs further investigation.

Changes in air quality, specifically higher levels of ground-level ozone and particulate matter are predicted to increase mortality from pulmonary and cardiovascular disease [4,9,54]. There is an estimated 0.01% increase in mortality from climate change-related elevated ground-level ozone concentrations [62]. Increased frequency and range of wildfires will add to mortality as air quality is compromised. Furthermore, burn injuries, PTSD, and acute exacerbation of respiratory conditions [3] and cardiovascular disease [11] have been associated with wildfire mortality.

Although our review did not find direct impact of increases in precipitation and flooding such as drowning events, the indirect impacts include disrupted access to health services [3,4,64]; mold [3] and other pathogen exposure [64]; failure to rescue related to disruption of services [4]; and risk of vector-borne disease [9,65].

There is variability in mortality risk for geographic locations of the U.S. and urban versus and rural communities. Southern regions of the U.S. seem to be better prepared for heat by physiological [21], behavioral [21], and structural factors [27,71] that influence overall heat adaptation [26,72,73,74]. In rural communities there are fewer heat programs and proportionally more outdoor workers within the population, thus increasing climate change mortality risk [77,79]. Drought is also a risk for rural communities as it has been associated with increases in mental health and suicidality [63,80]. Urban dwellers’ risk for heat mortality is influenced by UHIs caused by density of the built environment [71,77], and with an RCP of 8.5, mortality form UHIs is predicted to increase to 4.3% by 2100 [83].

Additional risks for climate mortality are related to gender, race and ethnicity, employment, and socioeconomic status. These are factors that are related to our national health disparities and are influenced by the determinants of health. These factors also cut to structural and social barriers for some groups within the U.S. population. Occupational risks of outdoor work and physical labor make men more vulnerable to climate change heat mortality [64,86,87,98] and more likely to die from accidents and injury related to climate change [39] and dehydration [28]. However, other studies found that gender risk is more closely related to cause of death [28] and race with non-White women having higher risk of death [35]. Hispanic males [88,98] and Black people were found to have higher risks of heat-related morbidity [88]. Hispanic workers also had higher mortality than Native American [98], and non-Hispanic workers in all industries [88]. Socioeconomic status, which is closely tied to occupation, racial and ethnic disparities, also influences climate change mortality as income affects adaptation to the environmental impacts of climate change [21,50,72].

There are limitations to this integrative review. Primarily, our review is limited to the published peer-viewed literature within the last decade (January 2010 thru May 2020) from the United States. The gray literature may offer additional insights to factors that influence and are responsible for climate change mortality. We limited our review to articles that specifically addressed factors that influence climate change-related mortality; however, climate factors can also influence morbidity that can lead to mortality such as heat-related illnesses that lead to increased hospitalization [96]. Additionally, the current method of tracking and reporting the cause of death most likely are unreported as the contributing factors for death may not be included in the report by the person completing the death certificate; heat-specific International Classification of Diseases (ICD) codes may underestimate heat-related mortality; and studies that rely on specific mortality (such as non-communicable disease (NCD) co-morbidities) are not sensitive enough to identify climate change-related mortality [88]. Furthermore, currently there are limited studies about wildfires and cardiovascular mortality [11] and there is not enough air monitoring in rural areas to support large studies of air quality and climate change mortality for rural populations. Despite the limitations, this review provides an examination of the factors that influence climate change-related mortality and the areas that need to be developed to fully realize the risk of climate change-related mortality in the United States.

## 5. Conclusions

This paper outlines the impact of climate change as a cause of death, whether from heat, cold, air pollution, precipitation, drought, wildfires, or infectious disease. Vulnerabilities that influence climate change-related mortality were noted to be age, gender, geographic setting, occupation, income level, and race and ethnicity. Our findings reflect the common indicators that influence morbidity and mortality in the U.S. [7]. The social determinants of health (gender, location, occupation, income, and race/ethnicity) along with physiological factors (age and morbidities) influence climate change-related mortality. These factors should be target areas when addressing climate change adaption and resilience.

Climate change has been identified as our greatest global health threat that will put the lives of people around the world at risk [107,108]. This review examined the factors that influence climate change-related mortality to describe the phenomenon in one country, the United States. Despite the importance of this issue to health of a nation, we found that capturing the full extent of climate change-related mortality has proved difficult. Relying on the published literature to identify the factors that influence climate change mortality may miss important findings that could be used to address policy and climate change adaptation programs. Depending on the state and the person completing the form, deaths certificates many only report the cause of death with a medical diagnosis, such as renal failure. The electronic health record (EMR) could be a valuable tool to search for climate change-related health indicators and mortality by linking ICD-10 codes. This would require some encouragement for providers to link climate, weather, and social indicators to the patient condition. There are classifications within the 2021 ICD-10 diagnosis codes for social issues (Z55-Z65), air pollution (Z77.110), flooding (X38.XXXA), heat (T67), and hypothermia (R68.0) but they are infrequently documented to record climate change-related mortality. Li and colleagues [92] used inpatient discharge data from ICD coding of the primary diagnosis, meteorological conditions, air pollution data, and ICD coded (by the investigators) death certificate to identify the impacts of extreme heat on climate change-related morbidity. Linking the ICD-10 codes that address climate change-related mortality to national weather and disaster information could further support accurate climate change mortality reporting. These data are important to provide the full impact of climate change to human health, for health care planning, to inform policy makers, and to promote climate adaptation and resilience plans for the U.S. population. An important next step would be to develop methods to quantify climate change-related mortality, instead of measuring impacts of single climate change events such as just wildfires or heatwaves. Quantifying climate change-related mortality as a whole can provide a comprehensive indicator of climate change impacts on the health of a population.

## Appendix A. List of Articles Included in the Review

**Reference Citation (Authors and Year with Corresponding Citation Number), Alphabetical**	**Climate Change Mortality Risk Factor(s)**	**Location of Population Studied**
Achakulwisut, P.; Anenberg, S.; Neumann, J.; Penn, S.L.; Weiss, N. 2019. [58]	Age, air quality, drought, geographic factors	Southwest United States
Acharya, P.; Boggess, B.; Zhang, K. 2018. [98]	Age, gender, heat, occupation, race/ethnicity	United States
Alexeeff, S.E.; Pfister, G.G.; Nychka, D. 2016. [60]	Air quality, geographic factors	United States
Anderson, G.B.; Bell, M.L. 2011. [18]	Heat, socioeconomic status (SES)	United States
Anderson, G.B.; Bell, M.L. 2012. [52]	Age, extreme weather, heat, power outages	New York City
Barnett, A.G.; Hajat, S.; Gasparrini, A.; Rocklöv, J. 2012. [42]	Extreme cold, heat, SES	United States
Barrett, B.; Charles, J.W.; Temte, J.L. 2015. [13]	Changing disease patterns, extreme weather, geographic factors, mental health, SES	Editorial. No location identified.
Bell, J.E.; Brown, C.L.; Conlon, K.; Herring, S.; Kunkel, K.E.; Lawrimore, J.; et al. 2018. [3]	Age, extreme weather (heat waves, floods, extreme precipitation, wildfires, droughts, hurricanes), geographic factors, race/ethnicity, SES	United States
Berman, J.D.; Ebisu, K.; Peng, R.D.; Dominici, F.; Bell, M.L. 2017. [63]	Drought	Western United States
Bobb, J.F.; Dominici, F.; Peng, R.D. 2011. [72]	Air quality	United States
Carnes, B.A.; Staats, D.; Willcox, B.J. 2014. [4]	Age, air quality, heat, rural/urban, water borne diseases	United States
Chang, H.H.; Zhou, J.; Fuentes, M. 2010. [62]	Air quality	Southeastern United States
Clearfield, M.; Pearce, M.; Nibbe, Y.; Crotty, D.; Wagner, A. 2014. [57]	Air quality, chronic diseases	United States
Conlon, K.C.; Rajkovich, N.B.; White-Newsome, J.L.; Larsen, L.; O’Neill, M.S. 2011. [43]	Age, extreme events (heat waves, floods, extreme precipitation, wildfires, droughts, hurricanes), geographic factors, race/ethnicity, SES	United States
Dominianni, C.; Lane, K.; Johnson, S.; Ito, K.; Matte, T. 2018. [44]	Extreme temperature (heat, cold), extreme weather events	New York City
Fang, Y.; Mauzerall, D.L.; Liu, J.; Fiore, A.M.; Horowitz, L.W. 2013. [104]	Air quality, geographic factors	Global
Fleischer, N.L.; Melstrom, P.; Yard, E.; Brubaker, M.; Thomas, T. 2014. [51]	Geographic factors, race/ethnicity, temperature (warmer cold seasons)	Alaska
Gronlund, C.J.; Cameron, L.; Shea, C.; O’Neill, M.S. 2019. [64]	Age, geographic factors, heat, precipitation	Michigan
Gronlund, C.J.; Zanobetti, A.; Schwartz, J.D.; Wellenius, G.A.; O’Neill, M.S. 2014. [27]	Age, heat	United States
Gubernot, D.M.; Anderson, G.B.; Hunting, K.L. 2015. [88]	Age, gender, heat, labor, race/ethnicity	United States
Guo, Y.; Barnett, A.G.; Tong, S. 2012. [89]	Age, heat	United States
Guo, Y.; Gasparrini, A.; Li, S.; Sera, F; Vicedo-Cabrera, A.M.; Tong, S. 2018. [33]	Geographic factors, heat	Global (multicountry)
Gutierrez, K.; LePrevost, C. 2016. [76]	Air quality, droughts, extreme weather, heat, race/ethnicity, rural/urban, SES	Southeastern United States
Hansel, N.N.; McCormack, M.C.; Kim, V. 2016. [20]	Air quality, temperature, geographic factors, urban/rural	No location identified
Harduar Morano, L.; Watkins, S.; Kintziger, K. 2016. [21]	Temperature, gender, race, geographic factors, age	Florida
Harlan, S.L.; Declet-Barreto, J.; Stefanov, W.L.; Petitti, D.B. 2013. [100]	Heat, race/ethnicity, rural/urban, SES	Arizona
Harlan, S.; Chowell, G.; Yang, S.; Petitti, D.; Morales Butler, E.; Ruddell, B.; et al. 2014. [28]	Age, gender, heat	Arizona
Hoehne, C.G.; Hondula, D.M.; Chester, M.V.; Eisenman, D.P.; Middel, A.; Fraser, A.M.; et al. 2018. [86]	Heat, geographic factors, SES, race/ethnicity, gender, age	United States
Isaksen, T.B.; Yost, M.; Hom, E.; Fenske, R. 2014. [90]	Heat, age	Washington
Isaksen, T.B.; Fenske, R.A.; Hom, E.K.; Ren, Y.; Lyons, H.; Yost, M.G. 2015. [97]	Heat, age	Northwestern United States
Jagai, J.S.; Grossman, E.; Navon, L.; Sambanis, A.; Dorevitch, S. 2017. [79]	Heat, geographic factors, rural/urban	Illinois
Johnson, M.G.; Brown, S.; Archer, P.; Wendelboe, A.; Magzamen, S.; Bradley, K.K. 2016. [87]	Heat, SES, gender, race/ethnicity, age	Oklahoma
Jones, B.; O’Neill, B.C.; McDaniel, L.; McGinnis, S.; Mearns, L.O.; Tebaldi, C. 2015. [5]	Extreme heat events, geographic factors, urban/rural	United States
Kalkstein, L.S.; Greene, S.; Mills, D.M.; Samenow, J. 2011. [40]	Heat, geographic factors, SES	United States
Kalkstein, A.J.; Kalkstein, L.S.; Vanos, J.K.; Eisenman, D.P.; Grady Dixon, P. 2018. [91]	Heat, heat waves, age	California
Kavouras and Chalbot. 2017 [54]	Temperature, air quality	United States
Kemble, S.K.; Lynfield, R.; DeVries, A.S.; Drehner, D.; Pomputius, W.F.; Danila, R. 2012. [66]	Infectious disease, heat, geographic factors	Minnesota
Kim, B.S.; Gavin, H.E.; Satchell, K.J.F.; D’Orazio, S.E.F. 2017. [67]	Infectious diseases/vector borne disease, heat	No location identified
Kingsley, S.L.; Eliot, M.N.; Gold, J.; Vanderslice, R.R.; Wellenius, G.A. 2016. [36]	Heat, race/ethnicity, SES, gender, occupation	Rhode Island
Klein Rosanthal, J.; Kinney, P.L.; Metzger, K.B. 2014. [105]	Heat, SES, age, race/ethnicity	New York City
Knapp, P.A.; Maxwell, J.T.; Ortegren, J.T. 2016. [69]	Temperature (heat waves and cold spells), geographic factors	55 U.S. Metro areas
Koman, P.D.; Romo, F.; Swinton, P.; Mentz, G.B.; de Majo, R.F.; Sampson, N.R.; et al. 2019. [22]	Heat, race/ethnicity, SES, geographic factors	Michigan
Lee, M.; Nordio, F.; Zanobetti, A.; Kinney, P.; Vautard, R.; Schwartz, J. 2014. [70]	Temperature, geographic factors	Unites States
Li, B.; Sain, S.; Mearns, L.O.; Anderson, H.A.; Kovats, S.; Patz, J.A. 2012. [92]	Heat	Wisconsin
Limaye, V.S.; Vargo, J.; Harkey, M.; Holloway, T.; Patz, J.A. 2018. [73]	Heat, age, geographic factors, SES	Eastern United States
Lin, S.; Hsu, W.-H.; Van Zutphen, A.R.; Saha, S.; Luber, G.; Hwang, S.-A. 2012. [84]	Temperature, extreme heat, age, gender, SES, race/ethnicity, urban/rural, geographic factors	New York State
MacFadden, D.R.; McGough, S.F.; Fisman, D.; Santillana, M.; Brownstein, J.S. 2018. [68]	Infectious diseases, heat	United States
Madrigano, J.; Ito, K.; Johnson, S.; Kinney, P.L.; Matte, T. 2015. [99]	Extreme temperature events (heat waves) (cold waves), race, geographic factors, SES	New York City
Madrigano, J.; Jack, D.; Anderson, G.B.; Bell, M.L.; Kinney, P.L. 2015. [85]	Heat, ozone, geographic factors, SES	Northeastern United States
Madrigano, J.; Mittleman, M.; Baccarelli, A.; Goldberg, R.; Melly, S.; Von Klot, S.; et al. 2013. [50]	Temperature (heat, cold), SES, age, geographic factors	Massachusetts
Matte, T.D.; Lane, K.; Ito, K. 2016. [37]	Heat, geographic factors, SES	New York City
Nordio, F.; Zanobetti, A.; Colicino, E.; Kloog, I.; Schwartz, J. 2015. [71]	Temperature-related mortality (heat/ warmer winters), SES, geographic factors	United States
Oleson, K.W.; Monaghan, A.; Wilhelmi, O.; Barlage, M.; Brunsell, N.; Feddema, J.; et al. 2013. [77]	Heat, urban/rural	United States and Southern Canada
Ostro, B.; Rauch, S.; Green, S. 2011. [29]	Heat	California
Parks, R.M.; Bennett, J.E.; Tamura-Wicks, H.; Kontis, V.; Toumi, R.; Danaei, G.; et al. 2020. [39]	Heat (injury deaths), gender, age	United States
Peng, R.D.; Bobb, J.F.; Tebaldi, C.; McDaniel, L.; Bell, M.L.; Dominici, F. 2011. [106]	Heat	Illinois
Petkova, E.P.; Horton, R.; Bader, D.; Kinney, P.L. 2013. [23]	Heat	Northeast United States
Petkova, E.P.; Bader, D.; Anderson, G.; Horton, R.; Knowlton, K.; Kinney, P.L. 2014. [17]	Heat	Pennsylvania, Ohio, Michigan, Minnesota, Oregon, Washington DC
Petkova, E.P.; Gasparrini, A.; Kinney, P.L. 2014. [6]	Heat, SES	New York City
Petkova, E.P.; Vink, J.K.; Horton, R.M.; Gasparrini, A.; Bader, D.A.; Francis, J.D.; et al. 2017. [38]	Heat, urban/rural	New York City
Post, E.S.; Grambsch, A.; Weaver, C.; Morefield, P.; Huang, J.; Leung, L.-Y.; et al. 2012. [61]	Air quality, geographic factors	United States
Raffa, R.B.; Eltoukhy, N.S.; Raffa, K.F. 2012. [65]	Disease and vector borne disease	United States
Reid, C.E.; Mann, J.K.; Alfasso, R.; English, P.B.; King, G.C.; Balmes, J.R. 2012. [30]	Heat	California, Massachusetts, New Mexico, Oregon, Washington
Reid, C.E.; Brauer, M.; Johnston, F.H.; Jerrett, M.; Balmes, J.R.; Elliott, C.T. 2016. [11]	Drought and wildfire, geographic factors, age	Australia, Portugal, Brazil, Canada, India, Greece, Russia, California, North Carolina
Riley, K.; Wilhalme, H.; Delp, L.; Eisenman, D. 2018. [101]	Heat	California
Roelofs, C. 2018. [102]	Occupation	California, Idaho, Texas, Kansas, Virginia, the “south”
Romeo Upperman, C.; Parker, J.; Jiang, C.; He, X.; Murtugudde, R.; Sapkota, A. 2015. [31]	Heat	United States
Rosen, J. 2016. [9]	Air quality, disease and vector borne disease, age	Discussion. No location identified.
Sampson, N.R.; Gronlund, C.J.; Buxton, M.A.; Catalano, L.; White-Newsome, J.L.; Parker, E.A. 2013. [103]	Heat, SES	Arizona, Michigan, New York, Pennsylvania
Schmeltz, M.T.; Marcotullio, P.J. 2019. [24]	Heat, floods, wildfires, vector-borne disease	United States
Schwartz, J.D.; Lee, M.; Kinney, P.L.; Yang, S.; Mills, D.; Sarofim, M.C.; et al. 2015. [46]	Winter Temperature	United States
Sheffield, P.E.; Landrigan, P.J. 2011. [94]	Vector-borne diseases, chronic disease, extreme weather, heat, air quality, SES	Global
Sheridan, S.C.; Allen, M.J. 2018. [74]	Geographic factors	Global, primarily developed countries
Sheridan, S.C.; Dixon, P.G. 2017. [93]	Age	United States
Sheridan, S.C.; Kalkstein, A.J. 2010. [80]	Geographic factors	United States
Shi, L.; Kloog, I.; Zanobetti, A.; Liu, P.; Schwartz, J.D. 2015. [47]	Heat, cold	Northeastern United States
Shi, L.; Liu, P.; Wang, Y.; Zanobetti, A.; Kosheleva, A.; Koutrakis, P.; Schwartz, J. 2016. [25]	Heat, winter temperature	Southeastern United States
Steinweg, C.; Gutowski, W.J. 2015. [19]	Heat	Missouri
Stone, B.; Vargo, J.; Liu, P.; Habeeb, D.; DeLucia, A.; Russel, A. 2014. [82]	Heat	Pennsylvania, Georgia, Arizona
Sun, J.; Fu, J.S.; Huang, K.; Gao, Y. 2015. [55]	Air quality	United States
Tagaris, E.; Liao, K.-J.; DeLucia, A.J.; Deck, L.; Amar, P.; Russell, A.G. 2010. [12]	Air quality	United States
Voorhees, A.S.; Fann, N.; Fulcher, C.; Dolwick, P.; Hubbell, B.; Bierwagen, B.; Morefeild, P. 2011. [34]	Heat	United States
Wang, Y.; Shi, L.; Zanobetti, A.; Schwartz, J.D. 2016. [48]	Cold	United States
Weinberger, K.R.; Haykin, L.; Eliot, M.N.; Schwartz, J.D.; Gasparrini, A.; Wellenius, G.A. 2017. [45]	Winter temperature; geographic factors	California, Georgia, Illinois, Florida, Massachusetts, New York, Pennsylvania, Texas, Washington DC
Weinberger, K.R.; Kirwa, K.; Eliot, M.N.; Gold, J.; Suh, H.H.; Wellenius, G.A. 2018. [49]	Heat, cold	Rhode Island, Massachusetts
Wilhelmi, O.V.; Hayden, M.H. 2010. [81]	Geographic factors	United States
Wilson, A.; Reich, B.J.; Nolte, C.G.; Spero, T.L.; Hubbell, B.; Rappold, A.G. 2017. [32]	Heat	United States
Wu, C.Y.H.; Zaitchik, B.F.; Gohlke, J.M. 2018. [53]	Heat	United States
Wu, J.; Zhou, Y.; Gao, Y.; Fu, J.S.; Johnson, B.A.; Huang, C.; et al. 2014. [75]	Geographic factors	Eastern United States
Xiao, J.; Peng, J.; Zhang, Y.; Liu, T.; Rutherford, S.; Lin, H.; et al. 2015. [26]	Heat, geographic factors	Eastern United States
Yang, T.-C.; Jensen, L. 2016. [14]	Geographic factors	United States
Yang, J.; Hu, L.; Wang, C. 2019. [78]	Cold, heat	United States
Yang, J.; Zhang, Y.; Wang, K.; Doraiswamy, P.; Cho, S.H. 2019. [56]	Air quality	United States
Zanobetti, A.; O’Neill, M.S.; Gronlund, C.J.; Schwartz, J.D. 2012. [95]	Heat, age, chronic disease	United States
Zanobetti, A.; O’Neill, M.S.; Gronlund, C.J.; Schwartz, J.D. 2013. [35]	Heat, gender	United States
Zhang, Y.; Xiang, Q.; Yu, C.; Bao, J.; Ho, H.C.; Zhang, L. 2019. [41]	Heat, cold (temperature variability)	United States, China, England
Zhang, Y.; Smith, S.J.; Bowden, J.H.; Adelman, Z.; West, J.J. 2017. [59]	Air quality	United States
Zhao, L.; Oppenheimer, M.; Zhu, Q.; Baldwin, J.W.; Ebi, K.L.; Bou-Zeid, E.; et al. 2018. [83]	Geographic factors	United States
